# Characterization of the Complete Mitochondrial Genomes of Two Sibling Species of Parasitic Roundworms, *Haemonchus contortus* and *Teladorsagia circumcincta*

**DOI:** 10.3389/fgene.2020.573395

**Published:** 2020-10-08

**Authors:** Nikola Palevich, Paul H. Maclean, Young-Jun Choi, Makedonka Mitreva

**Affiliations:** ^1^AgResearch Limited, Grasslands Research Centre, Palmerston North, New Zealand; ^2^McDonnell Genome Institute and Department of Medicine, Washington University School of Medicine, Saint Louis, MO, United States

**Keywords:** *Haemonchus contortus*, *Teladorsagia circumcincta*, mitochondrial genome, helminth, parasite, anthelmintic-susceptible

## Abstract

*Haemonchus contortus* and *Teladorsagia circumcincta* are among the two most pathogenic internal parasitic nematodes infecting small ruminants, such as sheep and goats, and are a global animal health issue. Accurate identification and delineation of Haemonchidae species is essential for development of diagnostic and control strategies with high resolution for Trichostrongyloidea infection in ruminants. Here, we describe in detail and compare the complete mitochondrial (mt) genomes of the New Zealand *H. contortus* and *T. circumcincta* field strains to improve our understanding of species- and strain-level evolution in these closely related roundworms. In the present study, we performed extensive comparative bioinformatics analyses on the recently sequenced complete mt genomes of the New Zealand *H. contortus* NZ_Hco_NP and *T. circumcincta* NZ_Teci_NP field strains. Amino acid sequences inferred from individual genes of each of the two mt genomes were compared, concatenated and subjected to phylogenetic analysis using Bayesian inference (BI), Maximum Likelihood (ML), and Maximum Parsimony (MP). The AT-rich mt genomes of *H. contortus* NZ_Hco_NP and *T. circumcincta* NZ_Teci_NP are 14,001 bp (A+T content of 77.4%) and 14,081 bp (A+T content of 77.3%) in size, respectively. All 36 of the typical nematode mt genes are transcribed in the forward direction in both species and comprise of 12 protein-encoding genes (PCGs), 2 ribosomal RNA (*rrn*) genes, and 22 transfer RNA (*trn*) genes. The secondary structures for the 22 *trn* genes and two *rrn* genes differ between *H. contortus* NZ_Hco_NP and *T. circumcincta* NZ_Teci_NP, however the gene arrangements of both are consistent with other Trichostrongylidea sequenced to date. Comparative analyses of the complete mitochondrial nucleotide sequences, PCGs, A+T rich and non-coding repeat regions of *H. contortus* NZ_Hco_NP and *T. circumcincta* NZ_Teci_NP further reinforces the high levels of diversity and gene flow observed among Trichostrongylidea, and supports their potential as ideal markers for strain-level identification from different hosts and geographical regions with high resolution for future studies. The complete mt genomes of *H. contortus* NZ_Hco_NP and *T. circumcincta* NZ_Teci_NP presented here provide useful novel markers for further studies of the meta-population connectivity and the genetic mechanisms driving evolution in nematode species.

## Highlights

- In-depth comparative and phylogenomic analyses of the recently published complete mitochondrial (mt) genomes of the New Zealand *H. contortus* NZ_Hco_NP and *T. circumcincta* NZ_Teci_NP field strains to improve our understanding of species- and strain-level evolution in roundworms.
- Analysis of the A+T rich and non-coding repeat regions of the complete *Haemonchus* and *T. circumcincta* mt genomes supports their potential as ideal markers for easy species- and possibly strain-level identification with high resolution for future studies.
- Phylogenetic relationships of complete mt genomes for all Trichostrongyloidea species currently available inferred from Bayesian, Maximum Likelihood and Maximum Parsimony analyses of mitochondrial genes, revealed high levels of intra-species diversity and a panmictic structure observed among Trichostrongylidea.
- Our findings indicate that a three-pronged approach incorporating phylogenetic inertia, pangenome structure/features and environmental data are needed in order to understand the mitochondrial genome evolution.

## Introduction

*H. contortus* (barber's pole worm) and *T. circumcincta* (brown stomach worm), are the most economically important pathogenic nematodes infecting small ruminants (sheep and goats) worldwide. These blood-feeding strongylid nematodes are orally transmitted via contaminated pasture to the host where they infect the fourth stomach (abomasum) reducing animal production and causing anemia, edema, and associated complications often leading to death (Vlassoff et al., 2001; Sutherland and Scott, 2010). Although these parasites can be managed using existing prophylactic drugs (anthelmintics), their remarkable natural tendency to develop resistance combined with the diminishing efficacy of compounds used, threatens the global livestock industry (Kaplan, 2004; Kaplan and Vidyashankar, 2012). While these monoxenous and obligately sexual species have identical life-cycles (Veglia, 1915), the gene flow and evolutionary relationships observed among the Trichostrongylidae superfamily, and the Strongylida suborder as a whole, remain unclear.

Parasitic roundworms belonging to the Haemonchidae family are able to infect a range of ruminant hosts. For example and on communal pastures in particular, *H. placei* is primarily a cattle parasite but can also infect small ruminants (Amarante et al., 1997; Gasser et al., 2008; Nunes et al., 2013), and vice versa for *H. contortus* (Jacquiet et al., 1998). Given the increase in availability of genomic information resources (Palevich et al., 2018), it is crucial for the management of parasitic diseases that readily accessible molecular tools are developed to enable end-users to easily determine which species is infecting which animal host.

The development of low cost, high-throughput, next-generation sequencing technologies have led to a recent focus on whole-genome sequencing (WGS) of complete nuclear genomes of parasitic roundworms (Nematodes) (Hu and Gasser, 2006; Hu et al., 2007; Jex et al., 2008; Palevich et al., 2019a,b), instead of earlier markers such as *cox1, cox2*, and *nad* genes. Owing to the substantial coverage following WGS (Wit and Gilleard, 2017; Palevich et al., 2019c) and advances in bioinformatics pipelines, further complete mitochondrial genome resources will be available to facilitate detailed comparative phylogenetic analyses across many species complexes and major taxonomic groups of nematodes (Hu et al., 2004; Gasser et al., 2016). In general, complete nematode mt genomes are ~12–21 kbp circular-DNA molecules (Hu and Gasser, 2006). Nematode mt genomes can provide rich sources of informative markers and typically feature (Wolstenholme, 1992; Boore, 1999; Saccone et al., 1999; Blouin, 2002): 12 protein-coding genes (PCGs) and lack of the *atp8* gene [except for *T. spiralis* (Lavrov and Brown, 2001)], 2 ribosomal RNA (*rrn*) genes (Gutell et al., 1993), and 22 transfer RNA (*trn*) genes (Wolstenholme et al., 1987); unidirectional transcription (usually in forward direction) with particularly unique initiation codons; and highly variable gene arrangements (Hu and Gasser, 2006). The gene sequences, and in particular the 12 PCGs, are relatively conserved are useful phylogenetic markers that have been used for examining phylogenetic relationships within the phylum Nematoda (Hu et al., 2004). The mitochondrial genetic code of nematodes follows translation (transl_table = 5)^1^ of National Center for Biotechnology Information (NCBI). In addition, nematode mt genomes also possess transposition genotypes of tRNAs that lack either a TΨC or DHU stem in the *trn* secondary structures (Wolstenholme et al., 1994; Yokobori, 1995). In the pursuit of improving the phylogenetic resolution within the phylum Nematoda, mt genomes serve as excellent markers for investigation of species delineation and the genetics of population evolution. Future efforts should focus on the availability of more complete mt genomes across all nematode species, and especially for conspecific strains.

For these reasons, this study interrogates the complete mitochondrial genome sequences of the anthelmintic-susceptible *H. contortus* NZ_Hco_NP (Palevich et al., 2019a) and *T. circumcincta* NZ_Teci_NP (Palevich et al., 2019b) derived from pasture-grazed New Zealand sheep. In this study, we performed numerous comparative and phylogenomic analyses to directly compare the molecular characteristics including the nucleotide composition and codon usage profiles of PCGs, as well as the secondary structures of each identified tRNA and rRNA gene within the two species, but also among other closely related trichostrongyloid nematodes. The phylogenetic position of *H. contortus* NZ_Hco_NP and *T. circumcincta* NZ_Teci_NP among Trichostrongylidae and of other species of socio-economically important parasites within the phylum Nematoda, are investigated based on mitochondrial nucleotide sequences and PCGs. This research provides insights into the mt genome evolution that may facilitate the development of molecular tools to differentiate between these two sibling species of parasitic roundworms at the strain level.

## Materials and Methods

### Sample Collection and DNA Extraction

The acquisition of parasite samples, DNA extraction, and genome sequencing technologies used to generate the mt genomes used for analysis in this study have recently been outlined in the form of brief reports (Palevich et al., 2019a,b). To fill any knowledge gaps associated with the above-mentioned reports, here we describe in detail the methodologies, sequencing technologies, and annotation software used to generate the *H. contortus* NZ_Hco_NP and *T. circumcincta* NZ_Teci_NP mt genomes. *H. contortus* and *T. circumcincta* were recovered from sheep infected with pure strains of parasites in Palmerston North, New Zealand, and total high molecular weight genomic DNA was extracted separately from multiple worms using a modified phenol:chloroform protocol, as previously described (Choi et al., 2017; Palevich et al., 2019a,b,c). DNA was deposited, stored and available upon request from AgResearch Ltd., Grasslands Research Center.

### Mitochondrial Genome Sequencing and Annotation

The complete mitochondrial genome sequences of *H. contortus* NZ_Hco_NP (Palevich et al., 2019a,c) and *T. circumcincta* NZ_Teci_NP (Palevich et al., 2019b) were obtained using next-generation sequencing technologies. The *H. contortus* NZ_Hco_NP (BioProject ID: PRJNA517503, SRA accession number SRP247265) whole genome shotgun paired-end (PE) and Single-Molecule, Real-Time (SMRT) long-read sequencing (SRA Runs: SRR11022845 and SRR11022846) were generated using the Illumina HiSeq2500 and Pacific Biosciences (PacBio) platforms, as previously described (Palevich et al., 2019a,c). The *T. circumcincta* NZ_Teci_NP (BioProject ID: PRJNA72569, SRA accession number SRP007648) whole genome shotgun library (SRA Run: SRR3145390) was generated as previously described (Tang et al., 2014; Palevich et al., 2019b), and sequenced by the Genome Center at Washington University (WUGSC, School of Medicine, St. Louis, MO, USA) using the Illumina MiSeq platform with the strategy of 150 bp paired-end sequencing mode. A total of 605 million raw reads were generated and made available in FASTQ format. The quality of the raw sequence reads was evaluated using the software package [http://www.bioinformatics.babraham.ac.uk/projects/fastqc (Andrews, 2010)]. The software Trimmomatic v.0.36 (Bolger et al., 2014) was used for removal of adapter, contaminant, low quality (Phred scores <30), and short (<50 bp) sequencing reads. The remaining high-quality sequencing reads were assembled *de novo* using the NOVOPlasty pipeline v.3.1 with default parameters and based on a kmer size of 39 following the developer's suggestions (Dierckxsens et al., 2016). The assembled mt genomes, tRNA and rRNA annotations were checked using the MITOS web server based on translation (transl_table = 5)^1^ (Invertebrate Mitochondrial) of NCBI (http://mitos.bioinf.uni-leipzig.de). Repeat-rich subsets of the mt genome assemblies were identified with varying degrees of complexity using the mDUST v.2006.10.17 (Morgulis et al., 2006) software tools with default settings. The sequence regions containing tandem repetitive elements were identified using Tandem Repeat Finder software online server (http://tandem.bu.edu/trf/trf.html). The coordinates of the found repeats were matched with coding sequences and the proportion of repeats overlapping coding regions was calculated. tRNA genes were further corroborated using the software SPOT-RNA (Singh et al., 2019) and the tRNA secondary structures were predicted using the ViennaRNA v.2.4 (Lorenz et al., 2011) and VARNA v.3.93 (Darty et al., 2009) packages. tRNA secondary structures and complete mt genomes were visualized using the Geneious Prime v.2019.1.1 (Kearse et al., 2012). The PCGs were manually curated by searching for ORFs (employing genetic code 5 as above) and alignment against the available *H. contortus* and *T. circumcincta* reference genomes in Geneious Prime v.2019.1.1 (Kearse et al., 2012).

### Mitochondrial Pangenome Analysis

The entire nucleotide sequences alignments using Mauve (Darling et al., 2004) were performed using Geneious Prime v.2019.1.1 (Kearse et al., 2012). Sequence differences among the *Haemonchus* and *Teladorsagia* species as well as between the respective strains was done using the *H. contortus* NZ_Hco_NP (Palevich et al., 2019a,c) and *T. circumcincta* NZ_Teci_NP (Choi et al., 2017; Palevich et al., 2019b) as the reference sequences, respectively. Repeated sequence elements of *Haemonchus* and *Teladorsagia* mt genomes were visualized in the form of Dotplots and generated based on pair-wise alignments with chaining coverage using PipMaker (Schwartz et al., 2000). Gene synteny was investigated using BLASTX alignments of sequences and genetic similarity mapping was visualized using the CGView Comparison Tool (http://stothard.afns.ualberta.ca/downloads/CCT) (Grant et al., 2012). For analysis of codon usage and amino acid composition, codon usage counts were extracted from each protein using Geneious Prime version 2019.1.1 (Kearse et al., 2012). These were then compiled and loaded into R version v.3.6.1 (R Core Team, 2013) where a clustered image map was produced using the “cim” function from the mixOmics package v.6.8.5 (Rohart et al., 2017).

### Phylogenomics Analysis of Complete mt Genomes

The phylogenetic positions of *H. contortus* NZ_Hco_NP and *T. circumcincta* NZ_Teci_NP among other species of nematodes available in the GenBank database were examined. The nucleotide sequences and PCGs of published Trichostrongyloidea mitochondrial genomes were retrieved from the National Center for Biotechnology Information, USA (http://www.ncbi.nlm.nih.gov/). *Caenorhabditis elegans* N2 (JF896456) was used as the outgroup species in this study. All coding sequences (CDS) used in this analysis had correct start and stop codons based on translation (transl_table = 5)^1^ of NCBI (Invertebrate Mitochondrial).

Considering the high degree of intraspecific variation in nucleotide sequences of mt genes of nematodes, we used the deduced amino acid sequences of mt proteins for phylogenetic analyses. Amino acid sequences from the 12 mt protein-coding genes of the complete mitochondrial genomes were deduced, aligned individually and concatenated into a single alignment using MAFFT v.7.450 (Katoh and Standley, 2013). Phylogenetic analyses were conducted using three methods: Bayesian inference (BI), Maximum Likelihood (ML), and Maximum Parsimony (MP) methods. BI analysis with the program MrBayes v.3.15265 (Huelsenbeck and Ronquist, 2001) and the MtInv model of amino acid evolution (Le et al., 2017) was selected as the most suitable model of evolution. However, since the MtInv model is not implemented in the current version of MrBayes, an alternative model, MtREV (Adachi and Hasegawa, 1996), was used in BI analysis. Four independent Markov chains were run for 1,000,000 metropolis-coupled MCMC generations, sampling a tree every 100 generations. The first 2,500 trees represented burn-in, and the remaining trees were used to calculate Bayesian posterior probabilities (PP). The evolutionary history was further inferred by using the ML and MP methods. ML analysis was performed with the MtREV General Reversible Mitochondrial + Freq. model (Adachi and Hasegawa, 1996) and MP was performed with PAUP^*^ v.4.0b10 (http://paup.csit.fsu.edu/) (Swofford, 2001). Both methods were calculated using 1,000 bootstrap replicates with bootstrapping frequencies (BF) were calculated. Phylograms were drawn using the software MEGA X (Kumar et al., 2018).

## Results and Discussion

### mt Genome General Features and Characteristics

The reconstruction of partial and complete mitochondrial genomes is becoming increasingly cost-effective with the advent of next-generation sequencing technology and recent advances in molecular parasitology tools, in particular for species identification and population genetics studies (Hu and Gasser, 2006; Jex et al., 2009). Despite these advances, only 12 species of Trichostrongyloid mt genomes are currently available in GenBank with only half of these represented by multiple strains (dos Santos et al., 2017). For these reasons, the complete mt genomes of New Zealand *H. contortus* NZ_Hco_NP (Palevich et al., 2019a,c) and *T. circumcincta* NZ_Teci_NP (Choi et al., 2017; Palevich et al., 2019b) field strains were sequenced and compared in detail to investigate the strain-level genetic differences of these two sibling Trichostrongyloidea species. Recently, complete mt genomes of NZ_Hco_NP and NZ_Teci_NP were generated using various sequencing platforms and assembled from 582 and 5.6 million (PE) reads with an average coverage depth per nucleotide of 13,293× and 2,097×, respectively.

**Figure 1:**
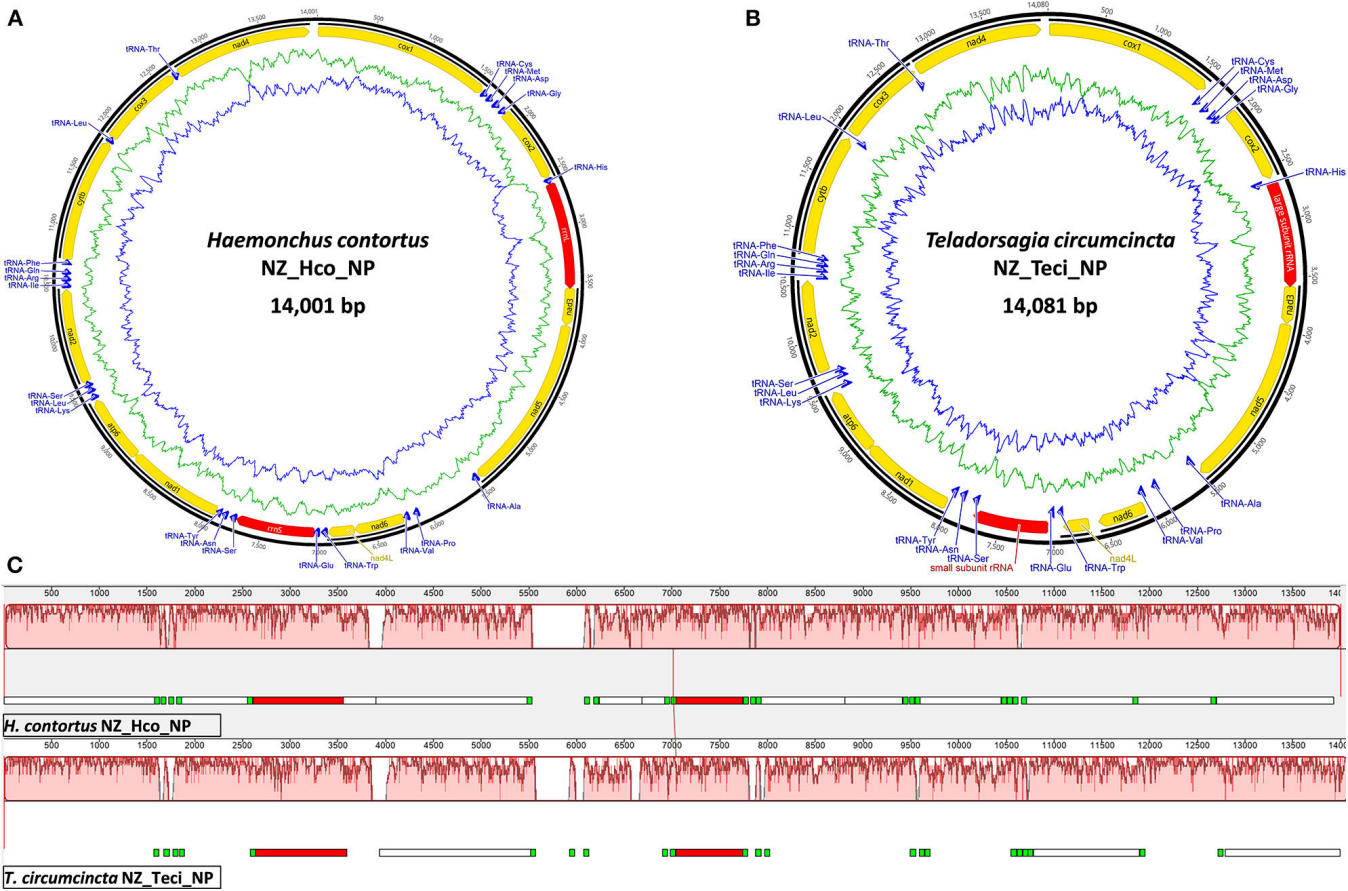
Mitochondrial genome atlases of *H. contortus* NZ_Hco_NP **(A)** and *T. circumcincta* NZ_Teci_NP **(B)**. Each map is annotated and depicts the 12 protein-coding genes (PCGs, yellow), two ribosomal RNA genes (*rrnS* and *rrnL*, red), 22 transfer RNA (*trn*, blue) genes, and any putative non-coding region if applicable (gray). The innermost circles depict GC (blue) and AT (green) content, respectively, along the genome. Mauve visualization of an alignment of the *H. contortus* NZ_Hco_NP (top) and *T. circumcincta* NZ_Teci_NP (bottom) complete mt genomes **(C)**. Mauve alignments of each genome are represented by a horizontal track, with annotated coding regions (white boxes), *trn* genes (green), and *rrn* genes (red). Red colored segments represent conserved regions among the two mt genomes.

The smallest Trichostrongyloidea mt genome published to date belongs to *Dictyocaulus* sp. cf. *eckerti* (Gasser et al., 2012) at 13,296 bp with the largest 15,221 bp mt genome belonging to *M. digitatus* (Jex et al., 2009). The NZ_Hco_NP and NZ_Teci_NP mt genomes are 14,001 and 14,081 bp in length, respectively (Figure 1) and are thus within the expected range. The reported mt genomes differ in size by ~16–54 bp than other *H. contortus* and *T. circumcincta* characterized strains, except for the 13.7 kb *H. placei* MHpl1 mt genome (dos Santos et al., 2017). The size discrepancies among the different strains primarily relate to A+T-rich control region expansions and longer non-coding regions between numerous transfer RNA genes.

**Figure 2:**
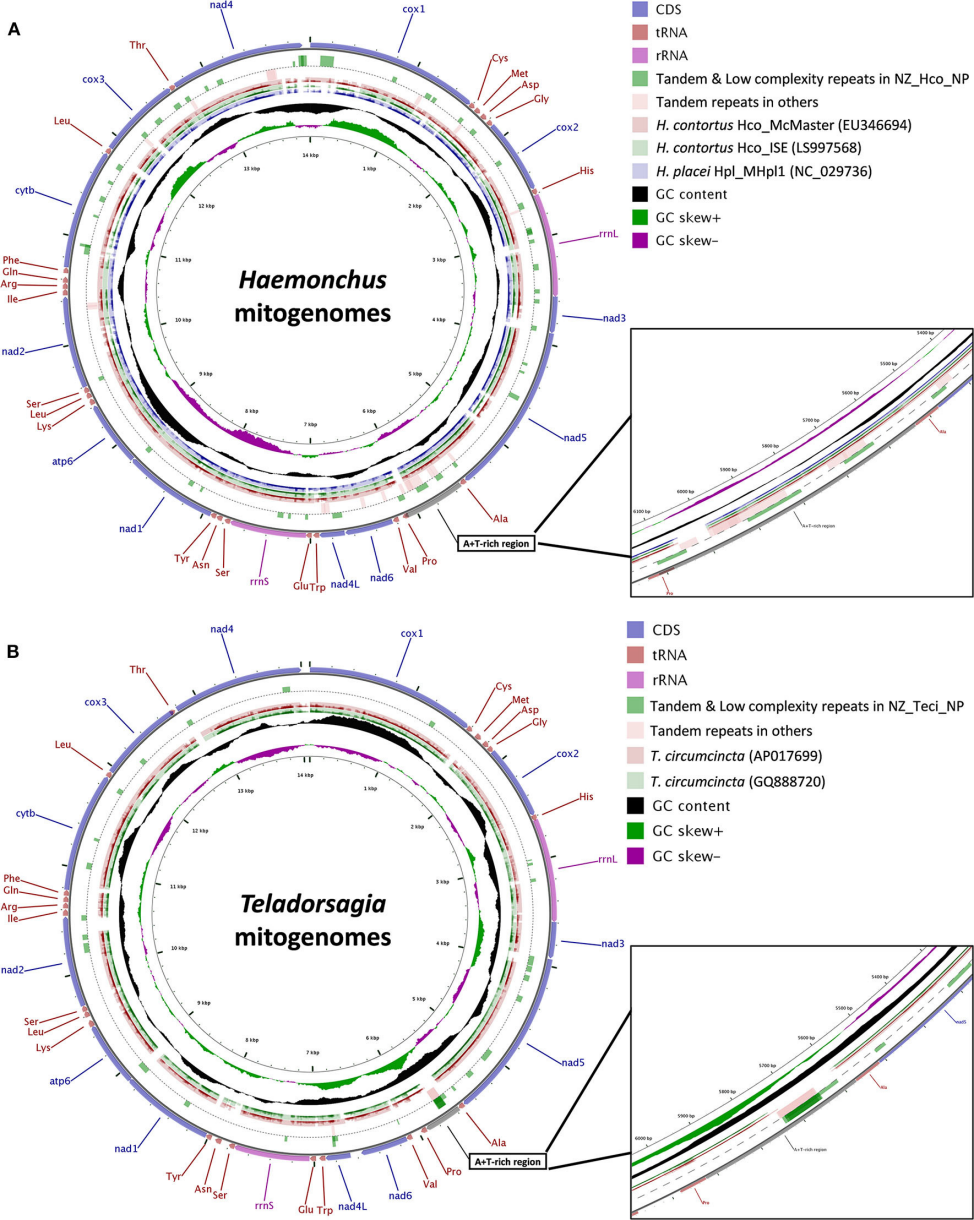
Gene arrangement and distribution of *Haemonchus* **(A)** and *Teladorsagia* **(B)** mt genomes. **(A)** Gene map of the complete mitochondrial genomes for *H. contortus* Hco_McMaster (light red, EU346694), *H. contortus* Hco_ISE (light green, LS997568) and *H. placei* Hpl_MHpl1 (light purple, NC_029736). **(B)** Gene map of the complete mitochondrial genomes for *T. circumcincta* AP017699 (light red) and GQ888720 (light green). *H. contortus* NZ_Hco_NP **(A)** and *T. circumcincta* NZ_Teci_NP **(B)** were used as the reference sequences, respectively. The outermost ring indicates gene arrangement and distribution based on the primary sequence GenBank file. Tandem repeat regions are shown with green blocks (full length) for *H. contortus* NZ_Hco_NP (circle 2) and light red blocks for all other *Haemonchus* mt genomes (circle 3). Low complexity repeat regions for *H. contortus* NZ_Hco_NP are also shown with green blocks (half length) on circle 2. The next two **(A)** or three **(B)** rings represent BLAST hits obtained from BLASTX searches performed using a threshold of 1e^−5^ for protein sequences of genomes described above, in which the query sequences were translated in reading frames 1 and 2. The inner-most rings indicate the GC content information. The contents of the A+T-rich regions have been shown in more detail in expanded 10× zoomed views. nad1-6, NADH dehydrogenase subunits 1–6; cox1-3, cytochrome c oxidase subunits 1–3; atp6, ATPase subunit 6; cytb, cytochrome b.

The total GC content of the *H. contortus* NZ_Hco_NP and *T. circumcincta* NZ_Teci_NP mt genomes were 21.1 and 22.8% and contained an A+T bias with an overall base composition of A = 32.9 and 31.2%, T = 44.5 and 46.1%, C = 6.3 and 7.3%, and G = 14.8 and 15.5%, respectively (Table 1). The overall A+T bias of 77.4 and 77.3% observed in *H. contortus* NZ_Hco_NP and *T. circumcincta* NZ_Teci_NP mt genomes, respectively, are within the typical range reported for nematode mitochondrial genomes (Hu et al., 2004; Hu and Gasser, 2006). Compared with the others, the A+T content of all *T. circumcincta* strains are within 0.1% of each other with *H. contortus* strains being ±0.5 of NZ_Hco_NP. Low-complexity regions (LCRs) are extremely abundant in eukaryotic proteins that are typically represented by amino acid sequences containing repeats of single amino acids or short amino acid motifs, and may facilitate the formation of novel coding sequences. *H. contortus* NZ_Hco_NP had 23 LCRs with 15 (65%) in coding regions, while *T. circumcincta* NZ_Teci_NP had 26 repeats with 21 (81%) found in coding regions (Figure 2), however determining the functional role of these repeats is a focus for future work. Comparative analysis of the *H. contortus* NZ_Hco_NP and *T. circumcincta* NZ_Teci_NP mtDNAs also revealed five and four tandem repeat (TR) regions, respectively (Figure 2).

**Table 1:** Comparison of the annotated mitochondrial genomes of *H. contortus* NZ_Hco_NP and *T. circumcincta* NZ_Teci_NP.

**Name**	**Type**	**Position**		**Length (bp)**	**Strand**	**Codons of PCGs**		**Nucleotide composition**							**Nucleotide skewness**	
		**Start**	**Stop**			**Start**	**Stop**	**T (%)**	**C (%)**	**A (%)**	**G (%)**	**AT (%)**	**GC (%)**	**GT (%)**	**AT skew**	**GC skew**
**Protein-encoding genes (PCGs)**																
*atp*6	Coding	8,809|8,901	9,408|9,500	600|600	+	ATT|ATT	TAA|TAA	46.3|46.7	6.3|6.7	29.5|30.8	16.3|15.8	75.8|77.5	22.6|22.5	62.6|62.5	−0.222|−0.205	0.442|0.404
*cox*1	Coding	1|7	1,582|1,578	1,582|1,572	+	ATA|ATA	TAA|TAA	42.7|43.8	9.7|10.9	26.5|25.1	19.6|20.3	69.2|68.9	29.3|31.2	62.3|64.1	−0.234|−0.271	0.338|0.301
*cox*2	Coding	1,862|1,898	2,554|2,593	693|696	+	ATA|ATT	TAG|TAG	42.6|43.7	7.8|9.8	30.4|28.0	17.5|18.5	73.0|71.7	25.3|28.3	60.1|62.2	−0.167|−0.219	0.383|0.307
*cox*3	Coding	11,880|11,959	12,648|12,759	769|801	+	ATA|ATT	TAA|TAA	46.8|46.1	7.4|7.3	24.7|31.2	17.4|15.5	71.5|77.3	24.8|22.8	64.2|61.6	−0.309|−0.193	0.403|0.36
*cyt*b	Coding	10,711|10,790	11,823|11,902	1,113|1,113	+	ATT|ATA	TAA|TAA	45.7|45.6	7.4|8.6	29.5|28.5	15.5|17.3	75.2|74.1	22.9|25.9	61.2|62.9	−0.215|−0.231	0.354|0.336
*nad*1	Coding	7,935|8,030	8,807|8,905	873|876	+	ATA|ATT	TAA|TAA	45.6|48.6	7.9|7.6	26.2|25.0	19.8|18.7	71.8|73.6	27.7|26.3	65.4|67.3	−0.27|−0.321	0.43|0.422
*nad*2	Coding	9,598|9,705	10,443|10,544	846|840	+	TTG|ATT	TAA|TAA	47.8|51.1	4.4|5.5	35.7|31.2	11.9|12.3	83.5|82.3	16.3|17.8	59.7|63.4	−0.145|−0.242	0.46|0.382
*nad*3	Coding	3,559|3,598	3,894|3,933	336|336	+	TTG|ATT	TAA|TAA	49.7|47.0	2.4|3.6	30.4|31.8	15.8|17.6	80.1|78.8	18.2|21.2	65.5|64.6	−0.241|−0.193	0.736|0.66
*nad*4	Coding	12,701|12,796	13,930|14,007	1,230|1,212	+	ATA|ATT	TAA|TAA	48.1|49.1	7.6|8.4	30.2|28.6	11.1|13.9	78.3|77.7	18.7|22.3	59.2|63.0	−0.229|−0.264	0.187|0.247
*nad*4L	Coding	6,688|6,672	9,919|6,903	232|232	+	ATT|ATT	TAA|TAA	53.0|57.3	2.6|2.2	29.3|26.7	13.8|13.8	82.3|84.0	16.4|16.0	66.8|71.1	−0.288|−0.364	0.683|0.725
*nad*5	Coding	3,896|3,942	5,477|5,520	1,582|1,579	+	ATT|ATT	TAA|TAA	44.5|48.8	6.3|7.3	32.9|30.0	14.8|13.9	77.4|78.8	21.1|21.2	59.3|62.7	−0.15|−0.239	0.403|0.311
*nad*6	Coding	6,241|6,135	6,681|6,572	441|438	+	ATT|ATT	TAA|TAA	46.3|52.5	4.3|6.2	29.7|27.4	19.3|13.9	76.0|79.9	23.6|20.1	65.6|66.4	−0.218|−0.314	0.636|0.383
**Transfer RNA**																
*trnA*	tRNA-Ala	5,478|5,521	5,534|5,578	57|58	+	GGG|GGG	CTA|TAA	42.1|37.9	7|8.6	40.4|37.9	10.5|15.5	82.5|75.8	17.5|24.1	52.6|53.4	−0.021|0	0.2|0.286
*trnR*	tRNA-Arg	10,514|10,620	10,569|10,674	56|55	+	AAA|AAA	TTT|TTT	37.5|43.6	8.9|7.3	41.1|36.4	12.5|12.7	78.6|80.0	21.4|20.0	50.0|56.3	0.046|−0.09	0.168|0.27
*trnN*	tRNA-Asn	7,821|7,879	7,876|7,938	56|60	+	TAA|TTA	TAA|AGT	41.1|41.7	5.4|6.7	35.7|31.7	14.3|20	76.8|73.4	19.7|26.7	55.4|61.7	−0.07|−0.136	0.452|0.498
*trnD*	tRNA-Asp	1,728|1,782	1,784|1,839	57|58	+	AAA|AAA	TAA|TAG	36.8|39.7	3.5|3.4	45.6|41.4	14|15.5	82.4|81.1	17.5|18.9	50.8|55.2	0.107|0.021	0.6|0.64
*trnC*	tRNA-Cys	1,583|1,579	1,636|1,633	54|55	+	ATT|ATT	ATT|TTT	40.7|45.5	3.7|5.5	46.3|40	9.3|9.1	87.0|85.5	13.0|14.6	50.0|54.6	0.064|−0.064	0.431|0.247
*trnQ*	tRNA-Gln	10,570|10,678	10,624|10,733	55|56	+	TGT|TTG	CAG|AAA	43.6|42.9	1.8|1.8	36.4|39.3	16.4|16.1	80.0|82.2	18.2|17.9	60.0|59.0	−0.09|−0.044	0.802|0.799
*trnE*	tRNA-Glu	6,988|6,990	7,042|7,047	55|58	+	GAG|GAG	TTG|TGT	38.2|41.4	3.6|3.4	43.6|37.9	14.5|17.2	81.8|79.3	18.1|20.6	52.7|58.6	0.066|−0.044	0.602|0.67
*trnG*	tRNA-Gly	1,807|1,845	1,861|1,898	55|54	+	AAT|ATT	TTA|TAA	41.8|42.6	3.6|5.6	45.5|37.0	7.3|14.8	87.3|79.6	10.9|20.4	49.1|57.4	0.042|−0.07	0.339|0.451
*trnH*	tRNA-His	2,556|2,592	2,610|2,646	55|55	+	AGC|AGC	CTA|TAT	32.7|38.2	3.6|5.5	43.6|36.4	16.4|20	76.3|74.6	20.0|25.5	49.1|58.2	0.143|−0.024	0.64|0.569
*trnI*	tRNA-Ile	10,455|10,552	10,513|10,612	59|61	+	ATT|ATT	ATA|TAG	39|39.3	6.8|4.9	42.4|42.6	11.9|13.1	81.4|81.9	18.7|18.0	50.9|52.4	0.042|0.04	0.273|0.456
*trnL1*	tRNA-Leu	9,489|9,596	9,543|9,652	55|57	+	GTT|GTT	ACT|CTA	29.1|33.3	9.1|7	43.6|43.9	18.2|15.8	72.7|77.2	27.3|22.8	47.3|49.1	0.199|0.137	0.333|0.386
*trnL2*	tRNA-Leu	11,824|11,904	11,879|11,958	56|55	+	GCA|TAC	GCT|ATT	33.9|41.8	5.4|3.6	46.4|43.6	8.9|10.9	80.3|85.4	14.3|14.5	42.8|52.7	0.156|0.021	0.245|0.503
*trnK*	tRNA-Lys	9,423|9,499	9,480|9,561	58|63	+	GTT|AAA	TTA|TAT	41.4|39.7	8.6|9.5	37.9|39.7	10.3|11.1	79.3|79.4	18.9|20.6	51.7|50.8	−0.044|0	0.09|0.078
*trnM*	tRNA-Met	1,649|1,683	1,705|1,742	57|60	+	AAT|AGT	TTA|TAT	33.3|31.7	10.5|13.3	35.1|33.3	21.1|21.7	68.4|65.0	31.6|35.0	54.4|53.4	0.026|0.025	0.335|0.24
*trnF*	tRNA-Phe	10,657|10,736	10,710|10,790	54|55	+	ATT|ATC	ATA|TAA	40.7|32.7	0.0|5.5	50|50.9	9.3|10.9	90.7|83.6	9.3|16.4	50.0|43.6	0.103|0.218	1|0.329
*trnP*	tRNA-Pro	6,085|5,932	6,140|5,988	56|57	+	CAA|CAA	TGA|GAA	35.7|42.1	5.4|1.8	39.3|43.9	19.6|12.3	75.0|86.0	25.0|14.1	55.3|54.4	0.048|0.021	0.568|0.745
*trnS1*	tRNA-Ser	7,744|7,748	7,798|7,803	55|56	+	AAA|TAA	TTT|TAT	41.8|39.3	3.6|7.1	43.6|39.3	9.1|14.3	85.4|78.6	12.7|21.4	50.9|53.6	0.021|0	0.433|0.336
*trnS2*	tRNA-Ser	9,544|9,652	9,597|9,705	54|54	+	AAC|AAT	TTT|TTA	38.9|44.4	13|13	29.6|25.9	16.7|16.7	68.5|70.3	29.7|29.7	55.6|61.1	−0.136|−0.263	0.125|0.125
*trnT*	tRNA-Thr	12,647|12,724	12,702|12,777	56|54	+	GTT|TGT	ACT|AAC	44.6|42.6	3.6|5.6	44.6|37	7.1|14.8	89.2|79.6	10.7|20.4	51.7|57.4	0|−0.07	0.327|0.451
*trnW*	tRNA-Trp	6,920|6,904	6,976|6,961	57|58	+	GTA|ATA	ATA|TAT	42.1|41.4	5.3|5.2	45.6|48.3	7|5.2	87.7|89.7	12.3|10.4	49.1|46.6	0.04|0.077	0.138|0
*trnY*	tRNA-Tyr	7,881|7,975	7,934|8,030	54|56	+	AAG|AAG	TTA|TGA	42.6|42.9	1.9|1.8	42.6|41.1	11.1|14.3	85.2|84.0	13.0|16.1	53.7|57.2	0|−0.021	0.708|0.776
*trnV*	tRNA-Val	6,180|6,081	6,234|6,135	55|55	+	AAA|AAT	TTT|CTA	41.8|40	1.8|5.5	49.1|47.3	7.3|7.3	90.9|87.3	9.1|12.8	49.1|47.3	0.08|0.084	0.604|0.141
**Ribosomal RNA**																
*rrnS*	Small subunit rRNA (12*S*)	7,041|7,050	7,743|7,749	703|700	+	TGT|AAG	TAA|TTA	38.0|41.0	5.8|7.4	41.8|36.0	13.5|15.6	79.8|77.0	19.3|23.0	51.5|56.6	0.048|−0.065	0.399|0.357
*rrnL*	Large subunit rRNA (16*S*)	2,611|2,638	3,561|3,599	951|962	+	TAA|AAA	TTG|CAT	40.7|43.3	5.3|5.5	42.2|39.7	10.7|11.4	82.9|83.0	16.0|16.9	51.4|54.7	0.018|−0.043	0.338|0.349
**Other features**																
AT-rich region	Non-coding	5,535	6,118	583	+	GAA	TTG	44.6	2.4	45.5	6.5	90.1	8.9	51.1	0.010	0.461
Full genome				14,001|14,081				44.5|46.1	6.3|7.3	32.9|31.2	14.8|15.5	77.4|77.3	21.1|22.8	59.3|61.6	−0.15|−0.193	0.403|0.36

*H. contortus NZ_Hco_NP|T. circumcincta NZ_Teci_NP*.

To date, mt genomes are available for many species of Trichostrongyloidea and the mt genomes of the trichostrongyloid *H. contortus* NZ_Hco_NP and *T. circumcincta* NZ_Teci_NP are in agreement, as they both comprise of 12 protein-encoding genes (PCGs), 22 transfer RNA (*trn*) genes, and 2 ribosomal RNA genes (*rrnS* or 12*S* ribosomal RNA and *rrnL* or 16*S* ribosomal RNA). The 12 mt PCGs of both *H. contortus* NZ_Hco_NP and *T. circumcincta* NZ_Teci_NP were encoded on the heavy strand and transcribed in the same direction. The order of genes observed in *H. contortus* NZ_Hco_NP and *T. circumcincta* NZ_Teci_NP are identical to the previously reported *H. contortus* (Palevich et al., 2018) and *T. circumcincta* (Jex et al., 2009), respectively (Figure 1). In accordance to the mt genomes of other Trichostrongyloidea, the reported mt genomes also all lack the *atp*8 gene. All of the 12 PCGs in both mt genomes are predicted to use unique translation initiation codons such as ATT (*atp*6, *cyt*b, and *nad*4-6), TTG (*nad*2-3), and ATA (*nad*1 and *cox*1-3) in *H. contortus* NZ_Hco_NP, with *T. circumcincta* NZ_Teci_NP predominantly using ATT and ATA (*cyt*b and *cox*1) (Table 1). Both mt genomes are predicted to use the complete termination codons TAA. Nevertheless, *cox*2 had a TAG translation termination codon similar to *nad*3 of *H. placei* where not all termination codons were complete stop codons, typical of nematodes (Jex et al., 2008; dos Santos et al., 2017).

Of the 22 predicted *trn* genes, size ranged from 54 to 63 bp in length (Table 1), in *H. contortus* NZ_Hco_NP and *T. circumcincta* NZ_Teci_NP mt genome sequences and are similar to those of other nematodes studied to date (Supplementary Figure 1). The characteristics associated with the secondary structures predicted for most tRNA genes include: a 7–8 bp acceptor stem (amino-acyl arm), a 5 bp anticodon stem and a T/U residue preceding. With the exception of *T. spiralis* (Lavrov and Brown, 2001), these characteristics are consistent with all previously published mt genomes for the Trichostrongyloidea (dos Santos et al., 2017) and Rhabditida [*C. elegans* (Blaxter et al., 1998)].

The *rrnS* (12*S*) and *rrnL* (16*S*) genes identified in the mt genomes of *H. contortus* NZ_Hco_NP (703 and 951 bp) and *T. circumcincta* NZ_Teci_NP (700 and 962 bp) were A+T biased (Table 1). Secondary structure models for the *rrn* genes (Supplementary Figure 2) of *H. contortus* NZ_Hco_NP and *T. circumcincta* NZ_Teci_NP were constructed based on models of nematodes that have been previously reported (Hu et al., 2004, 2007; Hu and Gasser, 2006). The *rrn* genes were particularly A+T rich with 79.8 and 77.0% for *rrnS* and 82.9 and 83.0% for *rrnL* in *H. contortus* NZ_Hco_NP and *T. circumcincta* NZ_Teci_NP, respectively. The overall base compositions of the *rrnS* genes were; A = 41.8 and 36.0%, T = 38.0 and 41.0%, C = 5.8 and 7.4%, and G = 13.5 and 15.6%, and that of the *rrnL* genes were; A = 42.2 and 39.7%, T = 40.7 and 43.3%, C = 5.3 and 5.5%, and G = 10.7 and 11.4%. Therefore, the A+T content of the *rrnS* and *rrnL* genes were comparable to the overall A+T content of both whole mt genomes. The *rrnL* gene in both *H. contortus* NZ_Hco_NP and *T. circumcincta* NZ_Teci_NP, is located between *trnH* and *nad*3. Similarly, the *rrnS* gene is located between *trnE* and *trnS1* almost 3.5 Kb from the *rrnL* and between *nad*4L and *nad*1 in both mt genomes (Figures 1, 2). The locations, sizes and secondary structure characteristics of the *rrnL* and *rrnS* genes are relatively conserved among all Trichostrongyloidea mt genomes analyzed, especially the stem regions of the *rrnL* genes between *H. contortus* and *T. circumcincta* strains.

### Comparative mt Pangenome Analysis of Trichostrongyloidea

Comparisons of the gene synteny as well as the nucleotide, codon usage and amino acid compositions revealed a highly conserved GA2 gene arrangement (*nad*6, *nad*4L, *rrnS, nad*1, *atp*6, *nad*2, *cyt*b, *cox*3, *nad*4, *cox*1, *cox*2, *rrnL, nad*3, *nad*5), and mt genome structure as previously reported for trichostrongyloids (Hu et al., 2004, 2007). Also, in accordance with previously published mt genomes the absence of the presumed non-essential ATP synthase F0 subunit 8 (*atp*8) gene reported only in *Trichinella spiralis* (Lavrov and Brown, 2001; Jex et al., 2008, 2009) and other *Trichuris* species such as *Trichuris ovis* and *Trichuris discolor* (Nematoda: Trichuridae), was absent in our data.

The predicted lengths of all the genes in *H. contortus* NZ_Hco_NP are very similar to those of *T. circumcincta* NZ_Teci_NP (≤1 amino acid difference). The sequence variability between *Haemonchus* species/strains and *T. circumcincta* strains of the Trichostrongyloidea superfamily was assessed by performing pairwise comparisons for each PCG of the mt genomes (Figure 2). Among the *Haemonchus* species/strains, the most variable genes were *nad*6 and *nad*2 with 13.8 and 10.2% of sequence divergence, respectively, and the most conserved genes were *cox*1, *atp*6, and *cox*2 with 93.6, 92.2, and 92.5% of sequence identity, respectively. For the *T. circumcincta* strains, we found that the most variable gene was *nad4* with 2.2% sequence divergence, with most genes being conserved at around 98.6% sequence identity. Based on our results, the most variable genes among Trichostrongyloidea nematodes were *nad*2, *nad*6, *nad*3, and *nad*5 with 56.1, 38.4, 23.1, and 21.3% of sequence divergence, respectively, with the most conserved genes were *cox*1, *atp*6, *cox*2, and *cox*3 with 87.9, 83.5, 85.3, and 86.9% of sequence identity, respectively. Pairwise comparisons of all the PCGs among Trichostrongyloidea nematodes and it was observed that the *nad4* gene was the most variable in terms of both the gene length and the sequence identity (Figure 2), where the predicted lengths differ by exactly 6 amino acids between *Haemonchus* (1,230 bp for *H. contortus* and *H. placei*) and *T. circumcincta* strains (1,212 bp). In comparison, the *nad*4 gene appears to be conserved among different Trichostrongyloidea species, for example *Trichostrongylus vitrinus* and *T. axei* (1,227 bp), while *M. digitatus, C. oncophora*, and *C. elegans* N2 all share 1,230 bp *nad*4 genes (Blouin et al., 1997; Blouin, 2002; Hu et al., 2004; Jex et al., 2009; Gasser et al., 2012; Xu et al., 2015).

A recent investigation comparing the divergences observed between ITS-1 and ITS-2 genetic nuclear markers with *cox*1 or *nad*4 genes (Blouin et al., 1997; Blouin, 2002), showed that mt DNA accumulates substitutions more quickly than nuclear genome markers and that *nad*4 genes in particular are a superior tool for prospecting species. For example, regarding *H. placei* and *H. contortus* mt genomes, a divergence in nuclear ITS-2 rDNA between these two species has previously been reported as 1.3% (Stevenson et al., 1995). Whereas, differences between nucleotide sequences of *H. placei* and *H. contortus* mt genome PCGs ranged between 10 and 20% (Gasser et al., 2006; dos Santos et al., 2017). Taken together, our findings are in agreement with previous reports suggesting the use of the most conserved mt *cox* genes for investigating systematics and speciation in nematodes (Hu et al., 2004; Hu and Gasser, 2006; Zarowiecki et al., 2007), while the variation observed in *nad* mt genes would be more appropriate for population genetics studies (Jex et al., 2009).

**Figure 3:**
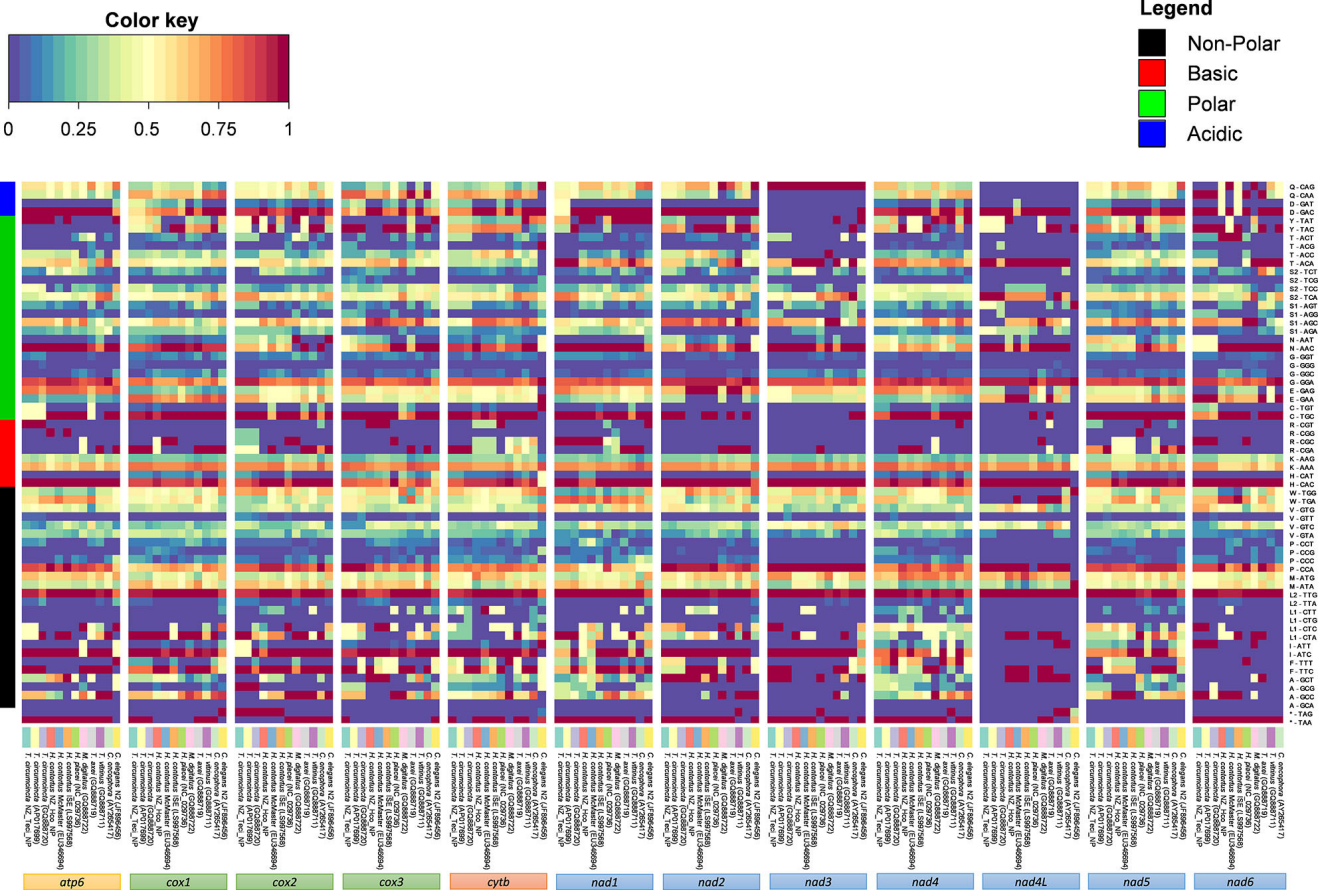
Amino acid and codon usage of Trichostrongyloidea complete mt genomes. Heat map analysis was performed based on relative abundances of protein sequence content determined for all mitochondrial genes. The relative inferred codon abundances per mt gene and specific to each mt genome are shown using a heat color scheme (blue to red), indicating low to high relative abundance. Mt genomes are groped based on previous phylogenomic comparison of concatenated PCGs. *C. elegans* N2 (GenBank accession number JF896456) was also included for comparison. Amino acid families are colored to represent non-polar in black, basic in red, polar in green, and acidic in blue.

Analysis of codon usage patterns in Trichostrongyloidea and *C. elegans* N2 revealed that certain codons are predicted to be utilized almost ubiquitously across all mitochondrial PCGs (Figure 3). As expected, the A+T bias reported in the Trichostrongyloidea mt genome sequences also affects the amino acid sequence composition of the predicted proteins. Within each codon family, we found that the non-polar Leu (TTG, 5.0%), basic His (CAC, 0.4%), polar Asn (AAC, 0.5%), and acidic Asp (GAC, 0.3% but except *nad3*) were highly utilized across all PCGs. Among each codon family, the T-ending (49.9%) codons are used most frequently than codons ending in any of the three other nucleotides, with C-ending (2.7%) codons least used. Similarly, for both Haemonchidae and Trichostrongylidae the average nucleotide content of their mt PCGs are 26.0% (A) and 43.7% (T), compared to 25.4% (A), and 44.2% (T), respectively. In particular the *nad*4L was the most T-rich at 53.5 and 54.9%, in each family, respectively. Also the most commonly codon observed was TAA as the termination codon with the majority of initiation codons also being AT-rich.

The observed nucleotide biases also have substantial effects on codon usage patterns for both *H. contortus* NZ_Hco_NP and *T. circumcincta* NZ_Teci_NP mt genomes (Table 2). The most frequently used codons among the PCGs were: TGT (Cys, *N* = 122 and 101 times used, 3.73% and 2.94% of the total), TTT (Phe, *N* = 435 and 428, 13.32% and 12.47%), ATT (Ile, *N* = 201 and 215, 6.15% and 6.26%), TTA (Leu, *N* = 199 and 271, 6.09% and 7.90%), TTT (Asn, *N* = 179 and 123, 5.48% and 3.60%), GTT (Val, *N* = 143 and 119, 4.40% and 3.50%), and TAT (Tyr, *N* = 181 and 198, 5.54% and 5.77%), respectively. In addition, T-ending codons are far more frequent than codons ending in any of the other three nucleotides among each codon family. For example, the least used codons included CCA (Pro, *N* = 3 and 14, 0.09% and 0.41%), GCG (Ala, *N* = 3 and 7, 0.09% and 0.20%), along with the C-ending codons GAC (Asp, *N* = 8 and 10, 0.25% and 0.29%) and CTC (Leu, *N* = 3 and 10, 0.09% and 0.29%).

**Table 2:** Codon usage analysis of PCGs in the mitochondrial genomes of *H. contortus* NZ_Hco_NP and *T. circumcincta* NZ_Teci_NP.

**AA**	**Codon**	**N**	**/1,000^a^**	**Freq**	**AA**	**Codon**	**N**	**/1,000^a^**	**Freq**
Ala	GCG	3|7	0.92|2.04	0.09|0.12	Pro	CCG	8|6	2.45|1.75	0.32|0.12
	GCA	12|10	3.67|2.91	0.38|0.18		CCA	3|14	0.92|4.08	0.12|0.27
	GCT	13|32	3.98|9.33	0.41|0.56		CCT	9|23	2.76|6.7	0.36|0.45
	GCC	4|8	1.22|2.33	0.13|0.14		CCC	5|8	1.53|2.33	0.2|0.16
Cys	TGT	122|101	37.35|29.44	0.84|0.84	Gln	CAG	13|18	3.98|5.25	0.3|0.37
	TGC	24|19	7.35|5.54	0.16|0.16		CAA	31|31	9.49|9.04	0.7|0.63
Asp	GAT	56|61	17.15|17.78	0.88|0.86	Arg	CGG	14|8	4.29|2.33	0.12|0.1
	GAC	8|10	2.45|2.91	0.13|0.14		CGA	6|1	1.84|0.29	0.05|0.01
Glu	GAG	17|32	5.21|9.33	0.3|0.43		CGT	15|21	4.59|6.12	0.12|0.26
	GAA	40|42	12.25|12.24	0.7|0.57		CGC	6|3	1.84|0.87	0.05|0.04
Phe	TTT	435|428	133.19|124.74	0.92|0.9		AGG	18|9	5.51|2.62	0.15|0.11
	TTC	36|46	11.02|13.41	0.08|0.1		AGA	62|38	18.98|11.08	0.51|0.47
Gly	GGG	25|43	7.65|12.53	0.26|0.3	Ser	AGT	69|70	21.13|20.4	0.41|0.43
	GGA	25|16	7.65|4.66	0.26|0.11		AGC	16|14	4.9|4.08	0.09|0.09
	GGT	38|72	11.64|20.99	0.4|0.5		TCG	20|10	6.12|2.91	0.12|0.06
	GGC	8|13	2.45|3.79	0.08|0.09		TCA	33|32	10.1|9.33	0.2|0.2
His	CAT	19|28	5.82|8.16	0.79|0.64		TCT	18|31	5.51|9.04	0.11|0.19
	CAC	5|16	1.53|4.66	0.21|0.36		TCC	13|6	3.98|1.75	0.08|0.04
Ile	ATA	133|103	40.72|30.02	0.37|0.31	Thr	ACG	11|15	3.37|4.37	0.15|0.17
	ATT	201|215	61.54|62.66	0.56|0.64		ACA	24|20	7.35|5.83	0.34|0.23
	ATC	27|19	8.27|5.54	0.07|0.06		ACT	24|48	7.35|13.99	0.34|0.55
Lys	AAG	49|37	15|10.78	0.3|0.27		ACC	12|5	3.67|1.46	0.17|0.06
	AAA	112|101	34.29|29.44	0.7|0.73	Val	GTG	47|42	14.39|12.24	0.18|0.17
Leu	TTG	128|160	39.19|46.63	0.32|0.29		GTA	59|62	18.06|18.07	0.22|0.26
	TTA	199|271	60.93|78.99	0.49|0.49		GTT	143|119	43.78|34.68	0.53|0.49
	CTG	11|17	3.37|4.95	0.03|0.03		GTC	19|18	5.82|5.25	0.07|0.07
	CTA	31|34	9.49|9.91	0.08|0.06	Tyr	TAT	181|198	55.42|57.71	0.84|0.82
	CTT	33|59	10.1|17.2	0.08|0.11		TAC	35|44	10.72|12.82	0.16|0.18
	CTC	3|10	0.92|2.91	0.01|0.02	Trp	TGG	44|48	13.47|13.99	1|1
Asn	AAT	179|123	54.81|35.85	0.85|0.9	Stop	TGA	32|44	9.8|12.82	0.14|0.16
	AAC	31|13	9.49|3.79	0.15|0.1		TAG	55|69	16.84|20.11	0.24|0.26
Met	ATG	52|56	15.92|16.32	1|1		TAA	142|154	43.48|44.88	0.62|0.58

*H. contortus NZ_Hco_NP|T. circumcincta NZ_Teci_NP*.

a *Represents number of codons per 1,000 codons*.

Our pairwise alignments and synteny analysis of the complete mt genome nucleotide sequences for the *Haemonchus* and *Teladorsagia* species and strains revealed a common and highly variable A+T-rich non-coding region between 5,500 and 6,100 bp in each mt genome (Figure 2 and Supplementary Figure 3). The A+T content of this region is unusually high in both *Haemonchus* (89.0%) and *Teladorsagia* (88.2%) mt genomes, and is located between *trnA* and *trnP* (or between genes *nad*5 and *nad*6). Among *Haemonchus*, the A+T-rich region is ~600 bp in *H. contortus* (89.7%) and 155 bp in *H. placei* (87.1%), whereas the region is 340 bp in *Teladorsagia* (88.2%). A pairwise alignment of the nucleotide sequences of the A+T-rich regions of *H. contortus* NZ_Hco_NP (611 bp) and closely related *H. contortus* McMaster (EU346694, 578 bp) resulted in 91.5% identity. Interestingly, the region in *H. contortus* is among the largest among other nematodes studied to date with the exception of *As. suum* (886 bp) (Okimoto et al., 1992; Keddie et al., 1998; Hu et al., 2003).

Comparative analysis of the control (A+T-rich) region of *Haemonchus* mtDNAs identified three tandem repeat (TR) units (69, 120, and 62 bp) compared to only one (126 bp) found in *T. circumcincta* strains mt genomes (Figure 2). The number of repeat units within the A+T-rich region was variable depending on the mtDNAs. For example, the copy numbers of the A+T-rich region TRs for *T. circumcincta* NZ_Teci_NP corresponding to TTATAATTATTATATAATAATTA (23 bp), TATATATATATAAATTAATATAATTATTATTAATAAT (37 bp), and ATATTATATATTATATATTA (20 bp) consensus units were 4, 2, and 4. In general, TRs of *Haemonchus* mt genomes were much more abundant and distributed randomly throughout the mtDNAs, whereas TRs were mostly in the intergenic regions of *T. circumcincta* mtDNA. Some TRs were found within the mitochondrial genes, such as those in *cox*1, *nad*5, *nad*6, and *nad*4, with also *nad*4L in *T. circumcincta* mtDNA. Overall, our analyses show that the number of tandem repeats was highly variable among different species and strains of *Haemonchus* and *Teladorsagia* and largely account for the variations among the mtDNA sequences lengths.

Based on our comparative genomics of A+T rich regions of complete *Haemonchus* species/strains and *T. circumcincta* strains mt genomes, we were able to differentiate individual strains with high confidence due to the high mutation rate and sequencing coverage for these regions. We propose that such regions can serve as ideal markers for future studies investigating nematode population structure (Jex et al., 2009; Gasser et al., 2012), population genetics (Blouin, 2002; Hu et al., 2004; Gasser et al., 2012), and phylogeny (Blouin et al., 1997; Blouin, 2002; Xu et al., 2015). For future work, we aim to design and test numerous primer pairs targeting the A+T-rich region between the *nad*5 and *nad*6 genes to develop accurate PCR-based molecular techniques to differentiate Trichostrongyloidea species members. Such molecular tools would enable easy species- and possibly strain-level identification with sufficient resolution and confidence from fecal samples that would also help prevent animal sacrifice for such studies.

### Mitochondrial Phylogenomics

**Figure 4:**
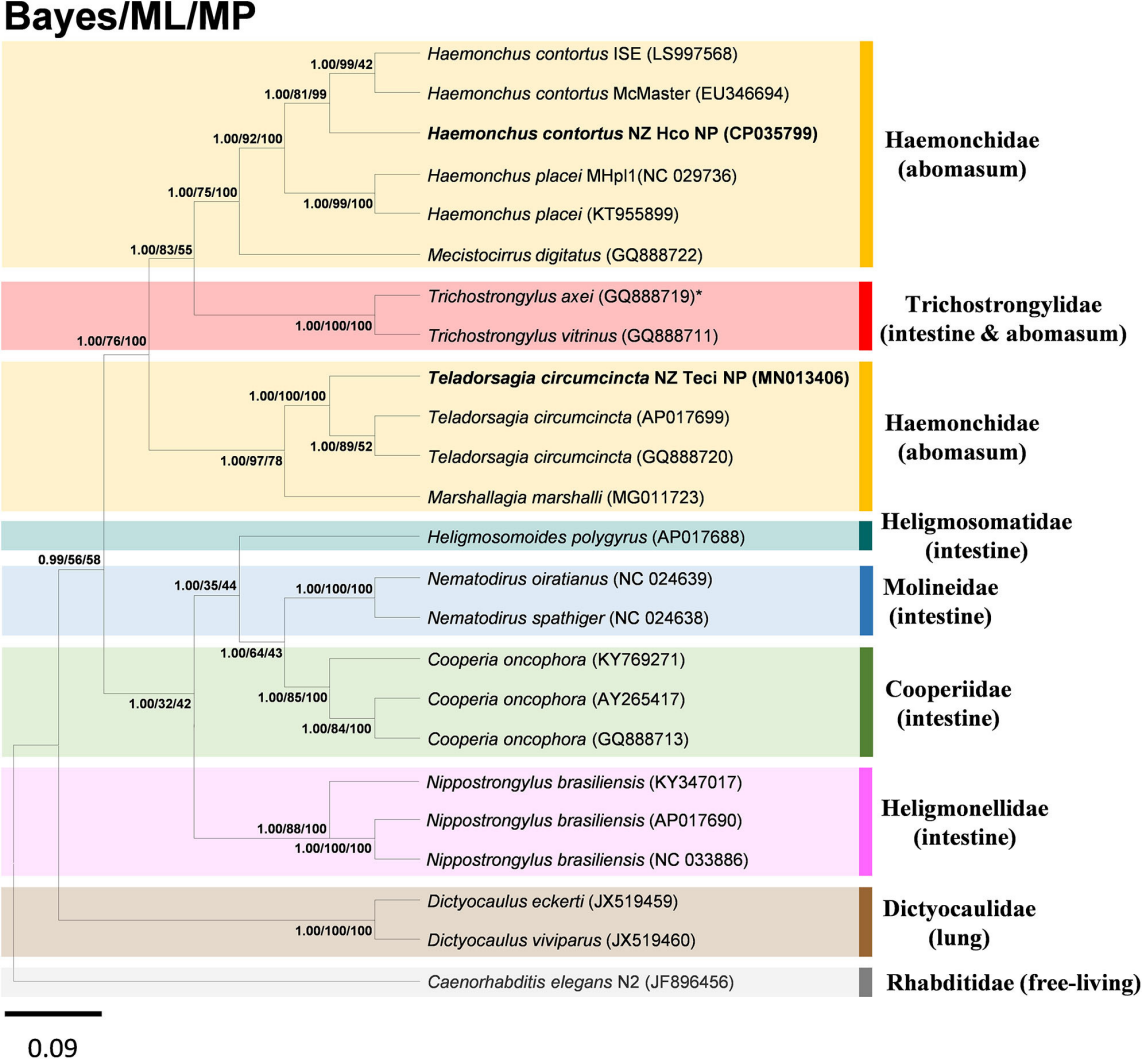
Inferred phylogenetic relationship among the nematode superfamily Trichostrongyloidea. Phylogenetic analysis of the complete mitochondrial genomes of different nematode species or conspecific strains is based on the concatenated amino acid sequences of 12 protein-coding genes by Bayesian inference (BI), maximum likelihood (ML), and maximum parsimony (MP) analyses. *H. contortus* NZ_Hco_NP and *T. circumcincta* NZ_Teci_NP are in bold, with *C. elegans* N2 (GenBank accession number JF896456) used as the outgroup. *species with different site of infection to other members of family. The numbers along branches indicate posterior probabilities and bootstrap values resulting from different analyses in the order: BI/ML/MP. Members of different families were shown in colored boxes with the associated main site of infection in parenthesis. GenBank accession numbers are provided (in parentheses) for all reference sequences. Scale bar represents Posterior Probability.

The super-family Trichostrongyloidea includes genera such as *Ostertagia, Teladorsagia, Trichostrongylus, Haemonchus, Cooperia, Nematodirus, Dictyocaulus*. To determine the phylogenetic relationship of the *H. contortus* NZ_Hco_NP and *T. circumcincta* NZ_Teci_NP with other *H. contortus* and *T. circumcincta* strains and members of the Trichostrongyloidea nematodes, the concatenated amino acid sequences predicted from 12 mtDNA PCGs were analyzed using BI, ML, and MP methods. Topologies of all trees inferred by the three different distance models and methods were identical (Figure 4), with phylogenetic relationships among the different Trichostrongyloidea species well-resolved with very high nodal support throughout. The presented phylogenomic tree of Trichostrongyloidea mt genomes, is consistent in topology with other published data (Chilton et al., 2006; Jex et al., 2009), and further support the hypothesis that the primary driver of early divergence is actually the site of infection in the host. Overall, rur results corroborate this hypothesis given that each of different species and strains of the Haemonchidae compared with Cooperidae and Trichostrongylidae families, are genetically closer to abomasal than to non-abomasal (intestine) Trichostrongyloidea, respectively. The anomaly to this hypothesis was *T. axei* that occupies the abomasum as the site of infection oppose to the small intestine of *T. vitrinus*, which implies the presence of other and yet uncharacterized drivers of genetic species diversity of these closely related parasites.

The phylogenomic tree based on the concatenated PCGs of the complete mt genomes (Figure 4), placed *H. contortus* NZ_Hco_NP and *T. circumcincta* NZ_Teci_NP in their respective clades with other *H. contortus* and *T. circumcincta* strains, which are in agreement with previous studies (Jacquiet et al., 1998; Nunes et al., 2013; Rohart et al., 2017; Palevich et al., 2018). However, our tree is not directly comparable to previously described phylogenetic trees that performed different analyses and compared a broader set of taxa, but mainly because the trees were based on the ITS rDNA nuclear genetic markers (Chilton et al., 2001; Hoberg et al., 2004; Jex et al., 2008). While both *Haemoncus* and *Teladorsagia* strains grouped together in their respective families, *T. circumcincta* NZ_Teci_NP was clearly separated from the other strains. Interestingly, *H. contortus* NZ_Hco_NP and McMaster (Australia) strains grouped together and originate from nearby geographic locations, but separate from *H. contortus* ISE (East Africa). This observation corroborates our hypothesis on the impact of environmental constraints and preferences acting as secondary drivers of within-species diversity. Overall, we are in agreement with previous phylogenetic studies suggesting that ITS rDNA nuclear genetic markers alone may not provide sufficient information to reveal higher taxonomic level relationships within the Trichostrongyloidea. Our findings indicate that a three-pronged approach that incorporates phylogenetic inertia, pangenome structure/features and environmental data in order to understand the mitochondrial genome evolution.

## Conclusions

This study compares the recently sequenced complete mitochondrial genomes of sibling species of parasitic roundworms, *H. contortus* and *T. circumcincta*, two of the most economically important and common pathogenic nematodes infecting small ruminants worldwide. We explored the mitochondrial pangenome features and phylogenomic relationships to assess species- and strain-level diversity among Trichostrongyloidea nematodes and with available mt genome in public databases. Our analyses corroborate previous studies showing that our *H. contortus* NZ_Hco_NP and *T. circumcincta* NZ_Teci_NP strains position in their respective clades. Future work should focus on utilizing our insights on the highly variable regions of *Haemoncus* and *Teladorsagia* conspecific strains to develop cost-effective DNA-based approaches for novel parasite management and control strategies. The complete mt genomes of the New Zealand *H. contortus* and *T. circumcincta* field strains are important contributions to our understanding of meta-population connectivity as well as species- and strain-level evolution in nematodes. With the continuing improvements in sequencing technology combined with a community effort we may be able to reconstruct the true origins of *Haemoncus* and *Teladorsagia*, as well as other parasitic nematodes that are of a global interest.

## Data Availability Statement

The datasets presented in this study can be found in online repositories. The names of the repository/repositories and accession number(s) can be found in the article/Supplementary Material.

## Author Contributions

NP conceived and designed the project, analyzed and interpreted the data, and wrote the manuscript. PM provided bioinformatics support for the project. MM and Y-JC provided resources and critically revised the manuscript. All authors read and approved the final manuscript.

## Conflict of Interest

NP was employed by the company AgResearch Limited. The remaining authors declare that the research was conducted in the absence of any commercial or financial relationships that could be construed as a potential conflict of interest.

## Footnotes

^1^https://ncbi.nlm.nih.gov/Taxonomy/Utils/wprintgc.cgi?chapter=tgencodes#SG5

## Supplementary Material

Supplementary Figure 1 Predicted secondary structures of the 22 tRNAs in the *H. contortus* NZ_Hco_NP (left) and *T. circumcincta* NZ_Teci_NP (right) mitochondrial genomes. The two-dimensional predicted layout predicted RNA secondary structures is shown with canonical base-pair (BP) in blue, and non-canonical, lone-pairs and triplets in green.

Supplementary Figure 2 Predicted secondary structures of the *rrn*S **(A)** and *rrn*L **(B)** genes in the *H. contortus* NZ_Hco_NP (left) and *T. circumcincta* NZ_Teci_NP (right) mitochondrial genomes. The two-dimensional predicted layout predicted RNA secondary structures were predicted based on the energy model of Mathews et al. (2004). The minimum free energy (MFE) structure of hairpins is colored according to the base-pairing probabilities (red, high; green, mid; blue, low). Blue and red circles around nucleotides represent the beginning and the end of molecules, respectively.

Supplementary Figure 3 Dot plots showing *Haemonchus* and *Teladorsagia* species and strain-level synteny. The horizontal and vertical axes represent the entire translated mt genome nucleotide sequences, respectively. Each aligning gap-free segment with more than 50% identity is plotted as a black line or dot. Analysis was performed using chaining coverage with PipMaker (Schwartz et al., 2000).
