# Supplementary figures and images for: Characterization of the Complete Mitochondrial Genomes of Two Sibling Species of Parasitic Roundworms, Haemonchus contortus and Teladorsagia circumcincta
Source: Front Genet. 2020 Oct 8;11:573395. doi: 10.3389/fgene.2020.573395 (PMC7578395; doi:10.3389/fgene.2020.573395)

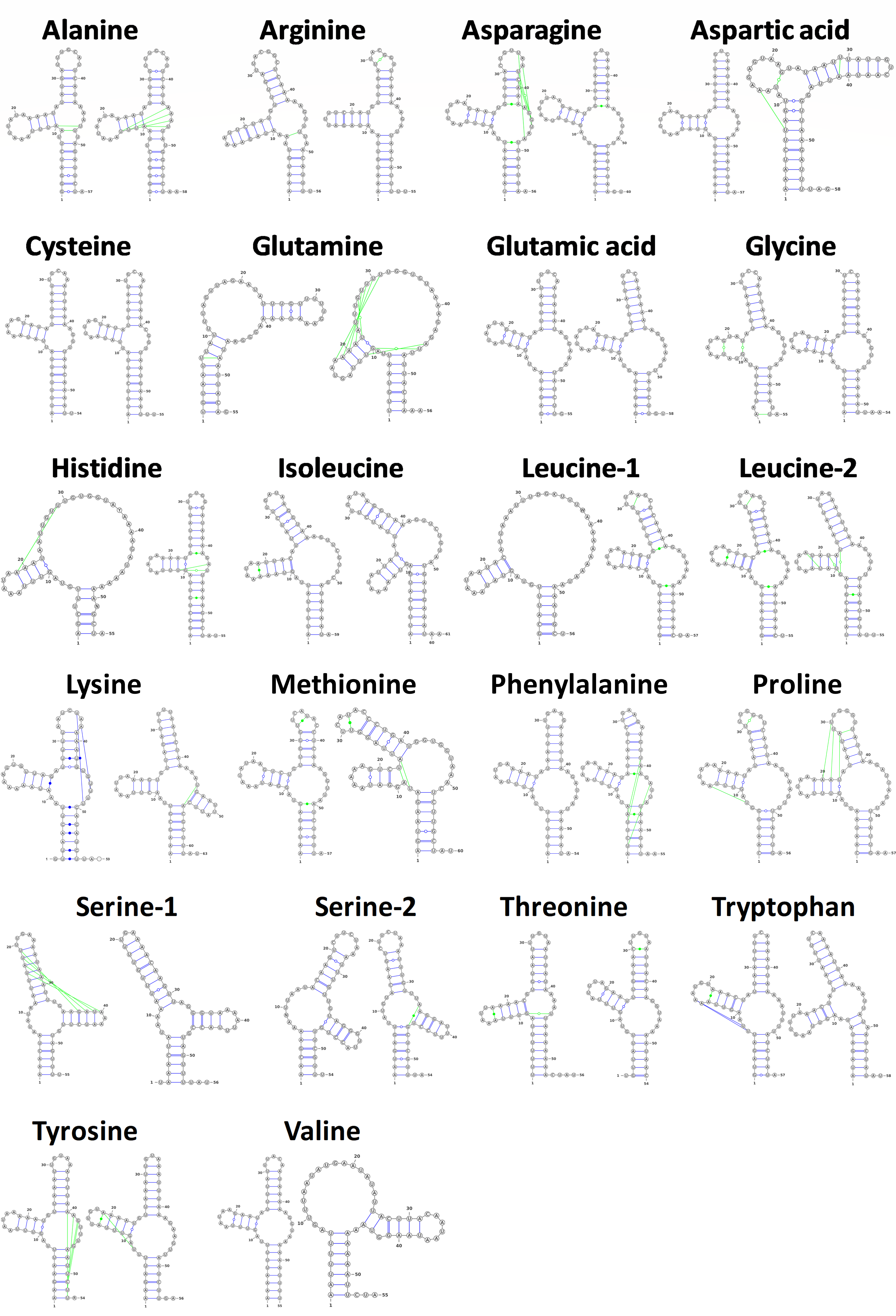

Supplement: Supplementary Figure 1 — Predicted secondary structures of the 22 tRNAs in the H. contortus NZ_Hco_NP (left) and T. circumcincta NZ_Teci_NP (right) mitochondrial genomes. The two-dimensional predicted layout predicted RNA secondary structures is shown with canonical base-pair (BP) in blue, and non-canonical, lone-pairs and triplets in green. [file Image_1.TIF]

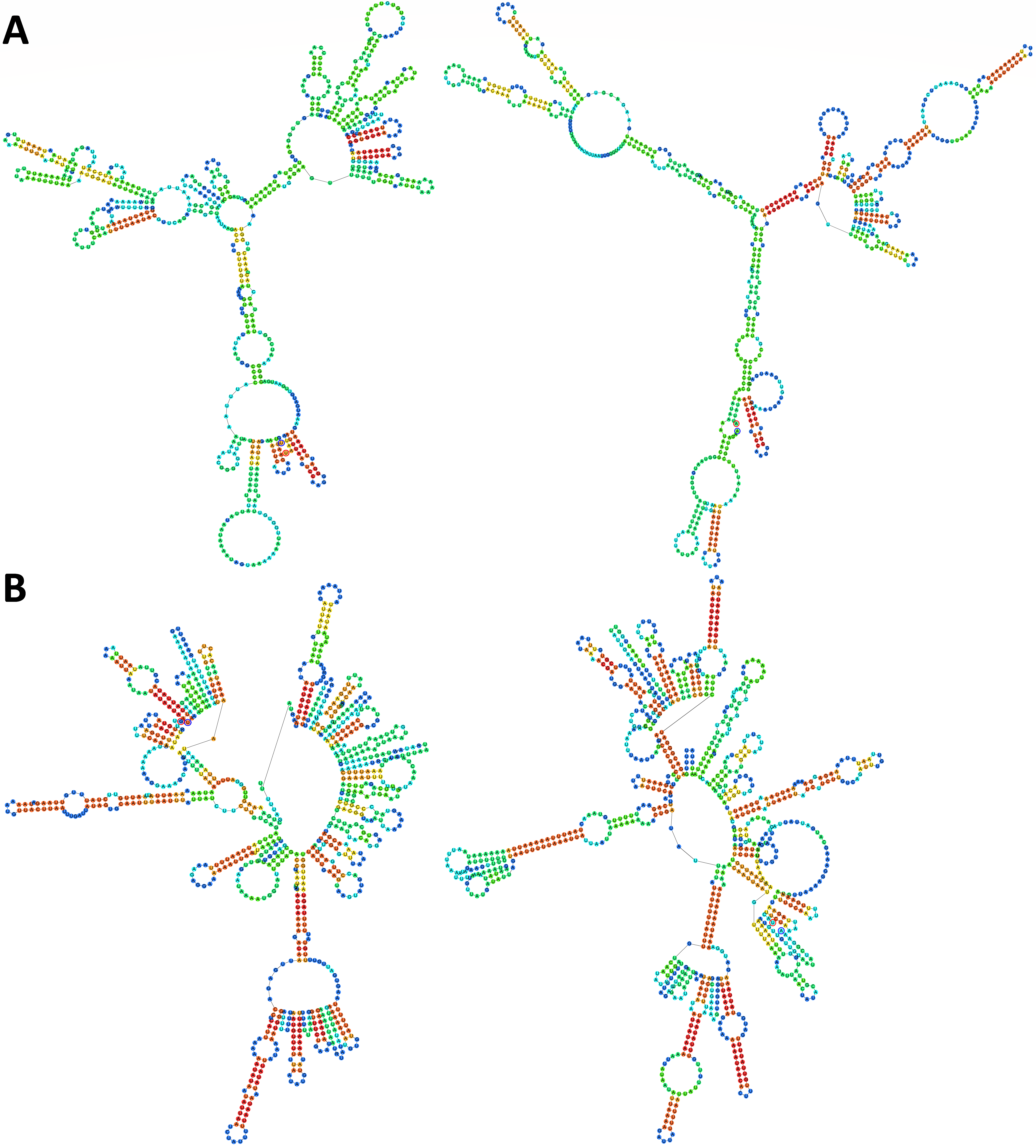

Supplement: Supplementary Figure 2 — Predicted secondary structures of the rrnS (A) and rrnL (B) genes in the H. contortus NZ_Hco_NP (left) and T. circumcincta NZ_Teci_NP (right) mitochondrial genomes. The two-dimensional predicted layout predicted RNA secondary structures were predicted based on the energy model of Mathews et al. (2004). The minimum free energy (MFE) structure of hairpins is colored according to the base-pairing probabilities (red, high; green, mid; blue, low). Blue and red circles around nucleotides represent the beginning and the end of molecules, respectively. [file Image_2.TIF]

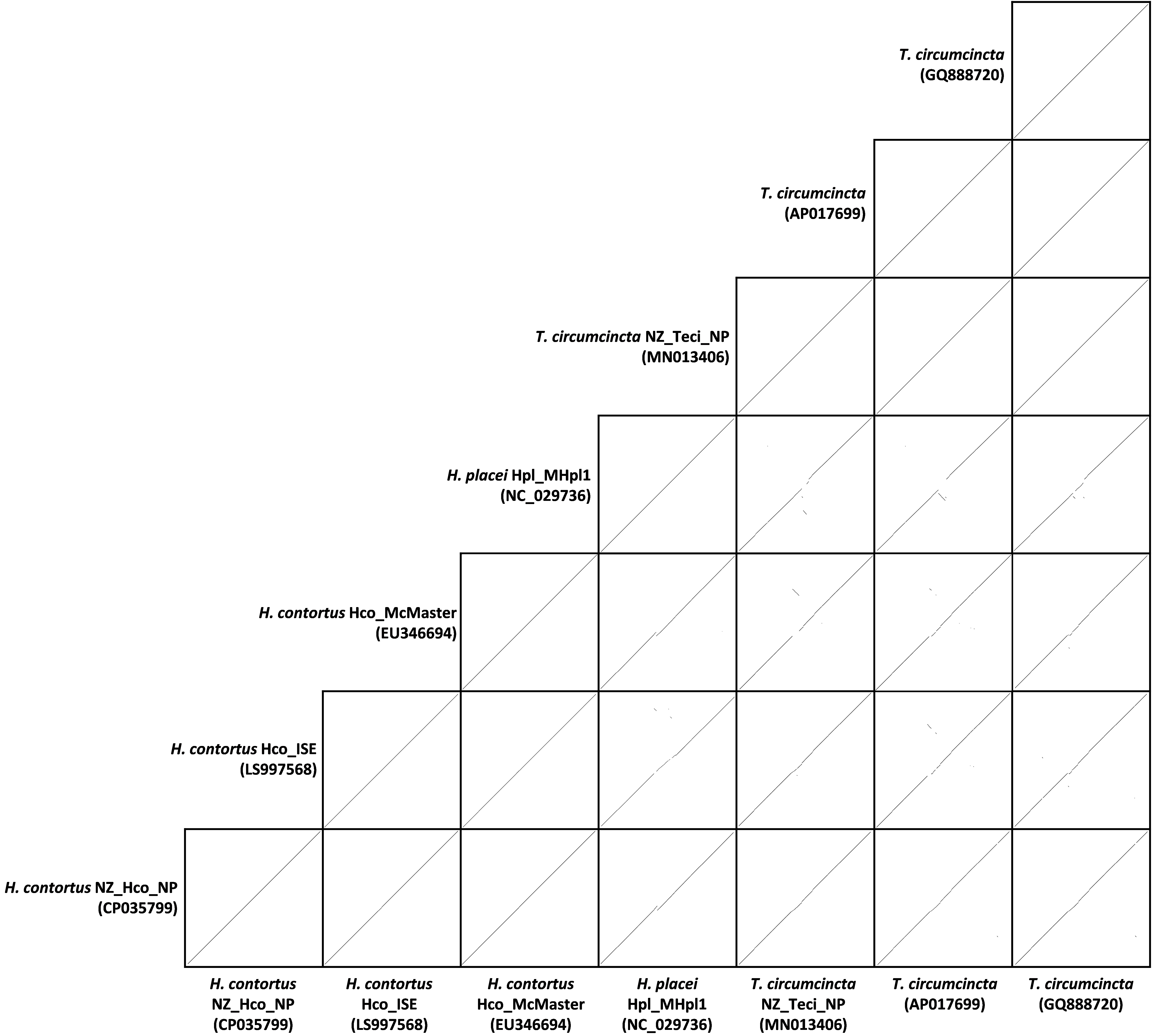

Supplement: Supplementary Figure 3 — Dot plots showing Haemonchus and Teladorsagia species and strain-level synteny. The horizontal and vertical axes represent the entire translated mt genome nucleotide sequences, respectively. Each aligning gap-free segment with more than 50% identity is plotted as a black line or dot. Analysis was performed using chaining coverage with PipMaker (Schwartz et al., 2000). [file Image_3.TIF]
